# The Potential of Digested Sludge-Assimilating Microflora for Biogas Production from Food Processing Wastes

**DOI:** 10.3390/microorganisms11092321

**Published:** 2023-09-15

**Authors:** Sato Hasaka, Saki Sakamoto, Katsuhiko Fujii

**Affiliations:** 1 Department of Chemistry and Life Science, School of Advanced Engineering, Kogakuin University, 2665-1 Nakano-cho, Hachioji 1920015, Tokyo, Japan; 2 Applied Chemistry and Chemical Engineering Program, Graduate School of Engineering, Kogakuin University, 2665-1 Nakano-cho, Hachioji 1920015, Tokyo, Japan

**Keywords:** anaerobic digestion, food waste recycling, methane, hydrogen, cellulase, pectinase, protease

## Abstract

Food processing wastes (FPWs) are residues generated in food manufacturing, and their composition varies depending on the type of food product being manufactured. Therefore, selecting and acclimatizing seed microflora during the initiation of biogas production is crucial for optimal outcomes. The present study examined the biogas production capabilities of digested sludge-assimilating and biogas-yielding soil (DABYS) and enteric (DABYE) microflorae when used as seed cultures for biogas production from FPWs. After subculturing and feeding these microbial seeds with various FPWs, we assessed their biogas-producing abilities. The subcultures produced biogas from many FPWs, except orange peel, suggesting that the heterogeneity of the bacterial members in the seed microflora facilitates quick adaptation to FPWs. Microflorae fed with animal-derived FPWs contained several methanogenic archaeal families and produced methane. In contrast, microflorae fed with vegetable-, fruit-, and crop-derived FPWs generated hydrogen, and methanogenic archaeal populations were diminished by repeated subculturing. The subcultured microflorae appear to hydrolyze carbohydrates and protein in FPWs using cellulase, pectinase, or protease. Despite needing enhancements in biogas yield for future industrial scale-up, the DABYS and DABYE microflorae demonstrate robust adaptability to various FPWs.

## 1. Introduction

Food waste represents the unnecessary biomass generated from the consumption of food in both households and industries. In 2019, 931 million tons of food waste were produced worldwide [1]. Of this, 86% is considered food garbage, a mixture of leftovers generated from households, restaurants, and retail stores [2]. The garbage is mainly composed of soluble sugars, starch, lipids, and proteins [3] and serves as a substrate for anaerobic digestion, a process that significantly reduces food waste volume and produces biogas [4]. Technologies for the anaerobic digestion of food garbage combined with physicochemical pretreatments have been extensively studied [5].

The remaining 14% of food waste is food processing wastes (FPWs), which are mainly composed of inedible parts generated during food manufacturing [2]. In developed countries, such as Japan and the United States, food manufacturers have managed to recycle approximately 95% of FPWs by using them as raw materials for composting or as animal feeds [6,7]. However, the number of farms is steadily declining in many countries [8], which suggests that this recycling rate may soon drop. Thus, novel uses for FPWs must be developed.

Anaerobic digestion is a bioprocess employed to break down organic waste, including garbage, sludge, and manure, and produces biogas—a renewable alternative to natural gas. This process involves sequential steps: hydrolysis, acidogenesis, acetogenesis, and methanogenesis, executed by a diverse group of hydrolase-producing bacteria, acidogens, acetogens, and methanogenic archaea, respectively [9]. Because anaerobic digestion can be employed to process a wide range of biomass wastes, it has received attention from the food industry regarding the recycling of FPWs for energy production, aiming to achieve zero emissions [10].

However, the technological approaches to anaerobically digesting FPWs are still in their nascent stages, with different challenges to those for food garbage. For instance, the rapid conversion of FPWs can lead to an overproduction of volatile fatty acids, resulting in increasing acid levels [11]. Additionally, the high protein content in FPWs leads to the accumulation of ammonia and hydrogen sulfide, making the digestion process unstable [11]. The composition of FPWs varies depending on the type of food product being manufactured. Thus, seed microflora used in the start-up of digesters should be specifically selected and acclimatized to ensure the successful digestion of FPWs [12]. Unlike food garbage, which generally consists of easily digestible materials such as starch, lipid, and protein, as described above, the major components of FPWs are cellulose, hemicellulose (from fruit and vegetable peels), and bone-derived collagen and hydroxyapatite [13]. Therefore, cultivating biogas-producing microflora that can effectively hydrolyze a wide range of FPWs may accelerate the acclimation period, reduce waste disposal costs, and conserve energy during food manufacturing.

We previously cultivated five sets of microflorae, two digested sludge-assimilating and biogas-yielding soil (DABYS) microflorae, by enriching riverbank soil, and three digested sludge-assimilating and biogas-yielding enteric (DABYE) microflorae, by enriching goat, sheep, and rabbit dung [14]. They can convert digested sludge, the stabilized residue from sewage sludge anaerobic digestion, into biogas. They primarily consist of representatives from the eubacterial genera, mainly *Clostridiaceae* and *Eubacteriaceae*, together with the *Methanobacteriaceae* family as major methanogenic archaea. Enzymatic analysis suggested that the microflorae could hydrolyze digested sludge through cellulase, chitinase, and protease activities. Heat-treated DABYS microflorae at 80 and 100 °C, which inactivated the methanogens but left thermotolerant bacteria unaffected, transformed methanogenic microflorae into hydrogenic ones [15]. Both *Clostridiaceae* and *Enterobacteriaceae* family bacteria are known to be involved in substrate hydrolysis and acido/acetogenesis during the anaerobic digestion of food garbage [16,17,18,19,20].

Considering the above-mentioned challenges limiting the successful conversion of FPWs into biogas, we aimed to assess the suitability of DABYS and DABYE microflorae as seed cultures for biogas production from FPWs, aiming for future applications in the food industry.

## 2. Materials and Methods

### 2.1. Chemicals and Materials

Standard gas of hydrogen, methane, and carbon dioxide were purchased from GL Sciences (Tokyo, Japan). Molecular biology reagents were purchased from Toyobo (Osaka, Japan), Thermo Fisher Scientific (Waltham, MA, USA), and MP Biomedicals (Santa Ana, CA, USA). All other chemicals were purchased from Wako Pure Chemical Industries (Kyoto, Japan). The glass and plastic labware used for enrichment culture were purchased from Maruemu Corporation (Osaka, Japan) and AS ONE Corporation (Osaka, Japan), respectively. The FPWs in Table 1 were supplied by grocery stores, restaurants, and food manufacturers in Hachioji city, Tokyo. In brief, cattle bone was obtained from a barbecue restaurant, and spent bonito flakes and spent dried kelp were from a Japanese-style restaurant. Spent tea leaves and spent coffee ground were obtained from a cafe. Soy sauce lees was provided by a soy sauce manufacturer. Fishbone (horse mackerel), peels of carrot, white radish, cabbage, lotus stem peel, apple, orange (*Citrus hassaku*), and grape (*Vitis* cv. Pione) were provided by the deli kitchen of grocery stores. Rice hull and rice bran were obtained from a rice mill plant, while wheat bran was provided by a flour milling company. Rapeseed oil cake was provided by a fertilizer manufacturer.

### 2.2. Preparation of Powders of FPWs

FPWs were dried completely in an FSP450 drying oven (Advantec, Tokyo, Japan) at 60 °C for 72 h (h), powdered using a WB-1 crusher (Osaka Chemical, Osaka, Japan), and sieved (1 mm pore). The sieved powder was used as a substrate for the enrichment culture. Extracts were prepared by autoclaving 1.0 g of each FPW powder suspended in 100 mL distilled water at 121 °C for 20 min. The total organic carbon (TOC) and total nitrogen (TN) contents in each extract were determined using a Shimadzu TOC-V CSH/TNM-1 analyzer (Shimadzu, Kyoto, Japan). The TOC, TN, and pH values of each FPW extract are listed in Table 1.

### 2.3. Seed Culture

Two types of DABYS and three types of DABYE microflorae were used as seed cultures to develop FPW-degrading and biogas-producing microflorae. DABYS microflorae enriched from two riverbank soils were designated as DABYS-A and DABYS-B, respectively. DABYE microflorae derived from the dung of goats, sheep, and rabbits were designated as DABYE-G, DABYE-S, and DABYS-R, respectively. Detailed descriptions of the sample locations are provided in the published article [14]. 

### 2.4. Vial-Scale Biogas Fermentation

Fermentation and subculture experiments were performed in 17 mL glass vials. For each vial, 100 μL of seed culture from each source (both DABYS and DABYE) was added to a mixture of 10 mL sterile water and 100 mg FPW powder. To create an anaerobic environment, the headspace in each vial was flushed with nitrogen for 1 min. Vials were sealed with butyl rubber stoppers and aluminum caps and incubated for 1 month (30 days) at 30 °C. After 1 month’s incubation, the headspace gas of each vial was sampled to quantify methane, hydrogen, and carbon dioxide yields. For subculturing, 100 μL aliquot from each vial was transferred to a fresh mixture of 10 mL sterile water and 100 mg FPW powder.

### 2.5. Biogas Analysis

The composition of the biogas was determined using a GC2014-TCD gas chromatography system equipped with a thermal conductivity detector (Shimadzu, Kyoto, Japan). The settings were as follows: Shincarbon ST column 50–80 (2.0 m × 3.0 mm internal diameter, Shinwa Chemical Industries, Kyoto, Japan); injection volume, 0.5 mL; injection port temperature 120 °C; carrier gas, argon (40.0 mL/min); column oven temperature, 120 °C; and the thermal conductivity detector, 260 °C. Peak identification and quantification of methane, hydrogen, and carbon dioxide in biogas was accomplished using standard pure gases of methane, hydrogen, and carbon dioxide gas, respectively (GL Science).

### 2.6. Enzymatic Assay

A 2.0 mL sample of the 1-month-subcultured microflora was centrifuged at 14,000× *g* and 4 °C for 10 min. The resultant supernatant, termed enzyme solution, was analyzed for the presence of cellulase, chitinase, and pectinase using the modified dinitro salicylic acid (DNS) method [21]. In brief, 200 μL of the enzyme solution and 800 μL of 50 mM citrate buffer (pH 4.8) containing 12.5 mg/mL of carboxymethyl cellulose, chitin from shrimp shells, or citrus pectin, as a substrate for cellulase, chitinase, or pectinase, respectively, were mixed in a microtube and incubated for 1 h at 50 °C. The tubes were then centrifuged at 14,000× *g* and 4 °C for 10 min. A total of 100 μL of the supernatant and 200 μL of the DNS reagent was incubated in boiling water for 5 min. After cooling to room temperature in an ice-water bath, 700 μL distilled water was added to the mixture, and the absorbance of the supernatant at 540 nm was measured using a UV-1800 spectrophotometer (Shimadzu). One unit of cellulase, chitinase, or pectinase activity was defined as the quantity of enzyme required to release 1 μmol of glucose, β-N-acetyl-D-glucosamine, or galacturonic acid via substrate hydrolysis in 1 h. Protease activity in the enzyme solution was quantified as the μg-trypsin equivalent activity using a Pierce protease assay kit (Thermo Fisher Scientific), according to the manufacturer’s instructions.

### 2.7. Community Fingerprinting of Microflorae

Microbial cells were collected from 1.0 mL of the subculture by centrifuging at 14,000× *g* and 4 °C for 10 min. Genomic DNA was extracted using the FastDNA SPIN Kit for Soil (MP Biomedicals). The 16S rRNA gene fragments corresponding to the V6–V8 region of eubacteria were amplified via polymerase chain reaction (PCR) using the primer pair F984GC/R1378 and the KOD Fx Neo polymerase (Toyobo), as described by Heuer et al. [22]. The PCR regimen comprised an initial denaturation at 94 °C for 2 min, followed by 34 cycles of 94 °C for 15 s, 50 °C for 30 s, and 68 °C for 30 s. For the amplification of methanogen 16S rRNA gene fragments corresponding to the V3 region, the nested PCR method was employed, as described by Zhou et al. [23]. The primary primer pair used was Met86f/Met915r for archaea, followed by the secondary primer pair GCARC344f/519r for methanogens. Amplification involved an initial denaturation at 95 °C for 5 min, followed by 30 cycles of 95 °C for 30 s, 56.5 °C for 30 s, and 68 °C for 30 s.

Denaturing gradient gel electrophoresis (DGGE) analysis of the PCR amplicons was performed using an NB-1480A DGGE system (Nihon Eido, Tokyo, Japan) according to the manufacturer’s instructions. Approximately 1 μg of each amplified product was loaded onto each well of a 6.0% polyacrylamide gel with a linear denaturant gradient of 50–70% for eubacterial amplicons and 35–45% for archaeal amplicons. Electrophoresis was performed at a constant voltage of 50 V for 18 h at 58 °C or 30 V for 17.5 h at 60 °C for an analysis of eubacterial or archaeal amplicons, respectively. The gels were stained using a solution of 10 μL of SYBR Green (Thermo Fisher Scientific) in 100 mL of TAE buffer (pH 8.0).

### 2.8. DNA Sequencing of the PCR-DGGE Amplicons

Distinct amplicons were visualized using a 470 nm light-emitting diode, and excised from the DGGE gels. The DNA was eluted by soaking the excised gel fragment containing the amplicon in 20 μL TE buffer (pH 8.0) for 72 h at 4 °C. Subsequently, 1.0 μL of the eluted DNA was used to reamplify rRNA gene fragments with a set of primers lacking the GC clamp [22,23,24] using the PCR regimen described above for DGGE analysis. The resulting PCR products were purified using the GeneJET PCR Purification Kit (Thermo Fisher Scientific) and directly sequenced using the BigDye Terminator v3.1 Cycle Sequencing Kit (Thermo Fisher Scientific). DNA sequences were compared to those of known microbial species in the GenBank, EMBL, and DDBJ databases using the BLAST algorithm.

### 2.9. Statistical Analysis

Statistical analyses for the experimental data were performed using Student’s *t*-test (*p* < 0.05).

## 3. Results and Discussion

### 3.1. Biogas Production from FPWs by the Subcultured Microflorae

Two DABYS and three DABYE microflorae were cultured with various FPWs suspended in water. The biogas yield from the 1st to the 10th subculture is detailed in Tables S1–S5 and Figure S1. All microflorae produced methane or hydrogen from most FPWs, except orange peel. Biogas production persisted through repeated subcultures. 

Microflorae produced methane from FPWs with low TOC/TN ratios (such as cattle bones, fish bones, and spent bonito flakes) and hydrogen from FPWs with high TOC/TN ratios (fruit peels, vegetable peels, and crop residues). DABYS-A produced 8.4–11.7 mL methane and up to 13.8 mL hydrogen per 1.0 g of respective FPWs (Table S1 and Figure S1a). DABYS-B exhibited similar biogas production characteristics, producing 9.0–14.3 mL methane and up to 15.8 mL hydrogen per 1.0 g of FPWs (Table S2 and Figure S1b). DABYS-G produced up to 14.2 mL hydrogen but only 3.6–8.0 mL methane from 1.0 g of FPWs, being less efficient in terms of methane production than DABYS-A and DABYS-B (Table S3 and Figure S1c). DABYS-S produced 3.7–7.5 mL methane and a maximum of 9.6 mL hydrogen from 1.0 g FPWs, not matching the yields of DABYS-A, DABYS-B, or DABYE-G (Table S4 and Figure S1d). DABYS-R was optimal for fermenting wheat bran and rapeseed oil cake, producing 17.5 mL and 15.1 mL hydrogen, respectively (Table S5 and Figure S1e).

As mentioned above, all microflorae were found to produce methane or hydrogen from various food residues whose pH and TOC/TN ratio ranged from 4.5 to 8.3 and 3.2 (nitrogen-rich) to 170.8 (carbon-rich), respectively. Therefore, we think that the microflorae acclimate to a wide variety of FPWs and use them as substrate for biogas production. For the successful anaerobic digestion of garbage, a mixture of different types of food wastes, its TOC/TN ratio is basic and serves as an important parameter. Since it is reported that a TOC/TN ratio of approximately 10–30 is suitable for the successful digestion of garbage [25,26], the microflorae seem to assimilate this and produce biogas.

Conversely, no microflora could produce biogas when fed with orange peels. Hence, their cultivation was aborted in the third subculture. Since citrus peel is known to contain limonene, an antibacterial monoterpene [27], our microflorae seem susceptible to this, and some pretreatment would be necessary to utilize orange peel for biogas production. For instance, biochar was recently found to be a suitable absorbent for limonene and its addition in digestion liquor successfully stimulated biogas production [28,29]. Some substrates, like lotus stem peel and rice hull, were fermented by the microflorae, but had lower gas yields than other FWPs.

Our microflorae could produce biogas at an ambient temperature, being energy-efficient. However, the biogas yields of our microflorae were lower than the standard rates reported in published articles. For instances, 350–380 mL of methane was produced from 1.0 g volatile solid of meat and bone meal [30], while 280 mL or 830 mL of methane was produced from 1.0 g volatile solid of defatted fish wastes (anchovy or salmon, respectively) containing heads, bones, and tails [31,32]. Agrawal et al. reported that 270–520 mL of methane is produced from 1.0 g solids of several fruit and vegetable peels [33]. While 120 mL or 50 mL of hydrogen was produced from 1.0 g of rice bran or wheat bran [34,35], respectively, 5.9 mL hydrogen was produced from 1.0 g of rice hull [36,37]. Approximately 800 mL of hydrogen was produced from 1.0 g of *Calophyllum inophyllum* oil cake [38], and 2.9–3.9 mL of hydrogen was produced from a 1.0 g volatile solid of acid–hydrolysate in coffee grounds [39]. Since the microflorae were found to show significant hydrolase activity, as mentioned below, the reason for the low biogas yield of our microflorae may be the increased gas pressure in the vial headspace [40,41] and lack of substrate pretreatment. Therefore, reducing the gas pressure and developing an optimal pretreatment method are indispensable for industrial-scale biogas production.

### 3.2. Eubacterial Compositions of the Subcultured Microflorae

**Figure 1:**
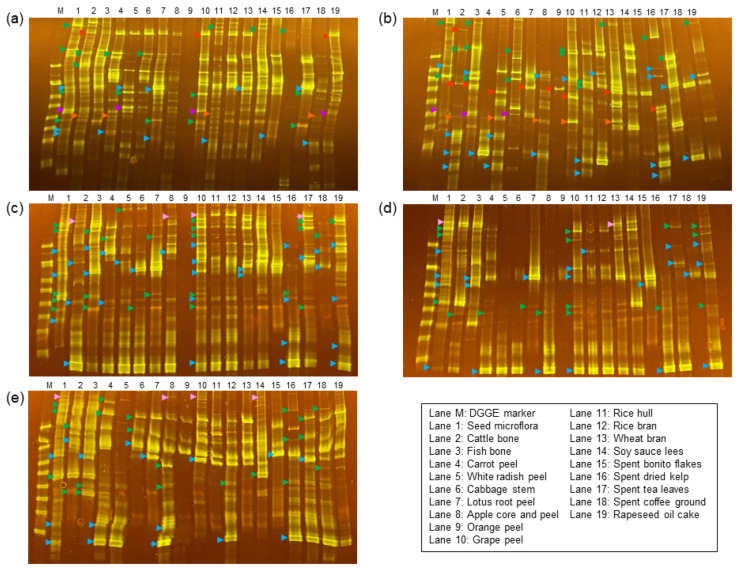
Denaturing gradient gel electrophoresis (DGGE) band patterns of eubacterial members from subcultured microflorae originating from DABYS-A (**a**), DABYS-B (**b**), DABYE-G (**c**), DABYE-S (**d**), and DABYE-R (**e**) seed microflorae. The data display patterns for the 10th subculture fed with FPWs, except for the third subculture fed with orange peel, along with their originating seed cultures. Green, blue, purple, red, orange, and pink arrowheads indicate major characteristic amplicons for the V6–V8 region of the 16S rRNA genes of the *Clostridiaceae*, *Enterobacteriaceae*, *Pseudomonadaceae*, *Bacillaceae*, *Lachnospiraceae*, and *Oscillospiraceae* families, respectively.

Eubacterial members play a role in substrate hydrolysis during anaerobic digestion [9]. To assess their genetic diversity, a DGGE analysis of the 16S rRNA gene fragments from subcultured microflorae was conducted. Within the subcultured microflorae samples, many rRNA gene amplicons were identified. Each of these fragments had a unique base sequence, which allowed them to be easily differentiated from one another. Significant shifts in eubacterial composition were observed when microflorae were exposed to FPWs compared to their original compositions (Figure 1). The DNA sequencing of major amplicon bands identified dominant members belonging to the *Clostridiaceae* (green arrowheads in Figure 1) and *Enterobacteriaceae* (blue arrowheads in Figure 1) families. Among the eubacterial species, some *Clostridiaceae* and *Enterobacteriaceae* strains were commonly found in both the subcultured florae and their seed florae, whereas other *Clostridiaceae* and *Enterobacteriaceae* strains, which were a minor population in their seed florae, were found to be dominant in the subcultured microflorae. *Pseudomonas* species, found in the DABYS seed microflorae and the corresponding subcultured flora (purple arrowheads in Figure 1), establish a symbiotic relationship with methanogens during anaerobic digestion. This promotes the syntrophic acetate oxidation and hydrogenotrophic methanogenesis (SAO-HM) pathway, preventing volatile fatty acid accumulation, which inhibits methanogenesis [42,43]. Conversely, *Bacillaceae* strains (red arrowheads in Figure 1) and *Lachnospiraceae* strains (orange arrowheads in Figure 1), despite initially being nearly undetectable in the seed florae, were predominant in the subcultured microflorae. DABYE-derived microflorae contained strains belonging to the *Oscillospiraceae* family (pink arrowheads in Figure 1), commonly found in herbivore gut microbiomes [44]. Thus, maintaining the diversity of eubacterial members in the original seed microflora appears to be important in the immediate acclimation to various FPW substrates and their hydrolysis, making it an ideal seed culture for biogas production from FPWs.

### 3.3. Archaea Compositions of the Subcultured Microflorae

**Figure 2:**
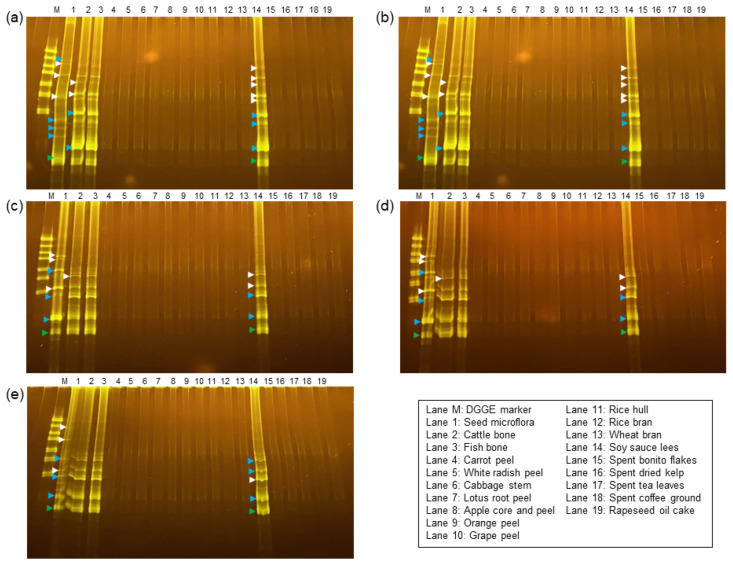
Denaturing gradient gel electrophoresis (DGGE) band patterns of methanogen members from subcultured microflorae originating from DABYS-A (**a**), DABYS-B (**b**), DABYE-G (**c**), DABYE-S (**d**), and DABYE-R (**e**) seed microflorae. The data display patterns for the 10th subculture fed with FPWs, except for the third subculture fed with orange peel, along with their originating seed cultures. Green, blue, and white arrowheads indicate characteristic amplicons for the V3 region of the 16S rRNA genes of *Methanobacteriaceae*, *Methanosarcinaceae*, and unidentified archaeal families, respectively.

Methane production from FPWs with a low TOC/TN ratio, such as cattle bone, fish bone, and spent bonito flakes, indicated the presence of methanogenic archaea in the subcultured microflorae. The *Methanosarcinaceae* (green arrowheads in Figure 2), *Methanobacteriaceae* (blue arrowheads in Figure 2), and an unidentified archaeal genus (white arrowheads in Figure 2), which were the major methanogens in the seed microflorae, were found to persist in the subcultured florae fed with low TOC/TN ratio FPWs. The unidentified genus includes only environmental clones, suggesting that they are unculturable. *Methanobacteriaceae* and *Methanosarcinaceae* methanogens are widespread in environments such as rice paddy soil, aquatic sediments, and anaerobic digesters [45,46,47]. These methanogens produce methane in two ways: *Methanosarcinaeae* strains utilize acetic acid to produce methane (acetoclastic pathway), while *Methanobacteriaceae* strains, being hydrogenotrophic, use hydrogen and carbon dioxide to yield methane [48]. These pathways can overlap; for example, the excess carbon dioxide generated in the acetoclastic pathway can be combined with hydrogen to synthesize methane [49]. Thus, both eubacterial and archaeal diversity may be beneficial for biogas production from various FPWs. However, when the microflorae were fed with high TOC/TN ratio substrates, most methanogenic archaea were not detectable, implying that dilution during repeated subcultures removed them.

### 3.4. Enzymatic Activity in Subcultured Microflorae

**Figure 3:**
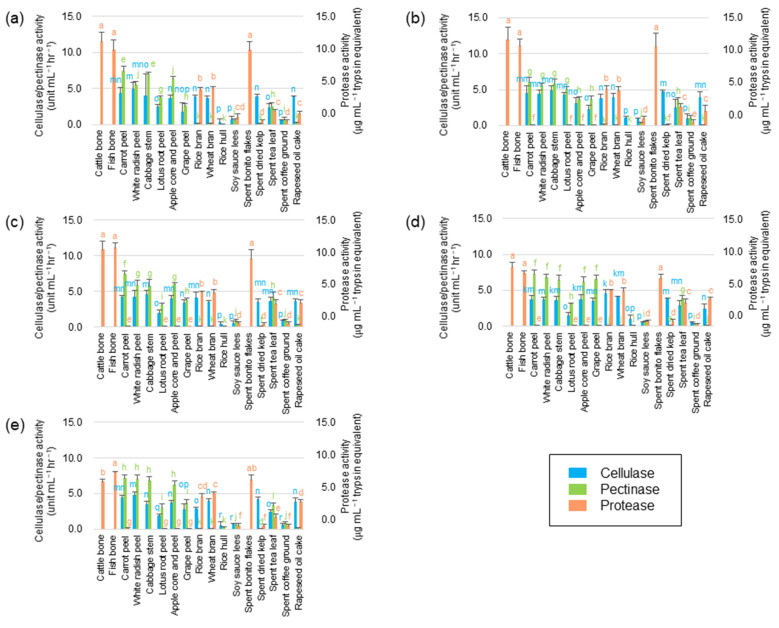
Cellulase, pectinase, and protease activities of the subcultures originating from DABYS-A (**a**), DABYS-B (**b**), DABYE-G (**c**), DABYE-S (**d**), and DABYE-R (**e**) seed microflorae. The data are presented as the mean ± standard deviation of independent triplicates. The blue-, green-, and orange-colored letters on the columns indicate significant differences at *p* < 0.05 (Student’s *t*-test) in cellulase, pectinase, and protease activity, respectively.

Substrate hydrolysis, driven by eubacterial hydrolase enzymes, kickstarts biogas production. Since FPWs mainly contain insoluble carbohydrates (such as cellulose), soluble carbohydrates (such as pectin), and protein [13], we examined the cellulase, pectinase, and protease activities of the subcultured microflorae. The results of the enzymatic assays of the tenth subculture of each microflora (excluding the microflora fed with orange peel) are illustrated in Figure 3.

Subcultures fed with low C/N ratio substrates (such as cattle bone, fish bone, or spent bonito flakes) exhibited protease activity, but lacked detectable cellulase and pectinase activities. Conversely, microflorae cultured with high C/N ratio substrates (vegetable and fruit residues) exhibited cellulase and pectinase activities with minimal protease activity, suggesting a change in eubacterial compositions despite originating from the same seed flora. The hydrolase activity of anaerobic digester microflorae is reported in several published articles. For instance, Jensen et al. reported that microflora fed with wheat straw showed 58–70 mg/L/h of MUF release from 4-methylumbelliferyl β-D-cellobioside, a substrate for their cellulase assay, which is comparable to 0.31–0.44 units/mL/h of cellulase activity determined by the DNS method [50]. Zhu et al. reported that microflora fed with corn straw possessed 95–120 units/L (which is comparable to 5.7–7.2 units/mL/h) of cellulase activity [51]. Mshandete et al. reported that microflora obtained from an anaerobic digester for potato processing wastes showed 0.3 units/mL/min (18 units/mL/h) of pectinase activity [52]. Parawira et al. reported that microflora obtained from an anaerobic digester for food processing wastes showed 0.003 units/mL/min (0.18 units/mL/h) pectinase and 0.008 units/mL/min (0.48 units/mL/h) of protease activity [53]. Gao et al. reported that microflora obtained from an anaerobic digester for kitchen wastes showed 0.04 units/mL/min (2.4 units/mL/h) of protease activity [54]. Through comparison with the published data, as shown above, our microflorae are suggested to possess significant hydrolase activity and have an advantage in the hydrolysis of FPWs. Microflorae subcultured with rice hull or lotus stem peels showed very weak hydrolase activity, which is consistent with their low biogas yields. Silica in rice hulls appear to inhibit the growth of hydrolase-producing bacteria in the microflorae [55], while antibacterial compounds in lotus stem may repress the growth of hydrolase-producing bacteria in the microflora [56].

DNA analysis highlighted the dominance of *Clostridiaceae* and *Enterobacteriaceae* families in the subcultured microflorae. Notably, certain bacterial families, including *Bacillaceae*, *Lachnospiraceae*, and *Oscillospiraceae*, which were minor in seed microflorae, flourished with FWPs. Both *Clostridiaceae* and *Enterobacteriaceae* families have species known for their cellulase, pectinase, and protease activities and hydrogen-producing potential [57,58,59,60,61,62], suggesting that they contribute to both FPWs hydrolysis and hydrogen production. Moreover, *Bacillaceae*, *Lachnospiraceae*, and *Oscillospiraceae* families are known for their proteolytic, pectinolytic, and cellulolytic activities, respectively [63,64,65,66]. Chitinase was not detected in any subculture, suggesting that chitinolytic strains diminished. 

Overall, DABYS and DABYE seed microflorae exhibit adaptability to fermentation substrates by changing their bacterial composition, in which FPW-specific hydrolytic and hydrogenic bacteria are enriched, and ultimately optimize FPW conversion to biogas.

## 4. Conclusions

In the present study, we subcultured DABYS and DABYE microflorae to convert FPWs into biogas, aiming for improved food waste recycling. The subcultured microflorae demonstrated the ability to hydrolyze carbohydrates and protein in FPWs, by using enzymes such as cellulase, pectinase, or protease. This facilitates methane or hydrogen production by respective microflorae. The results highlight the adaptability of DABYS and DABYE microflorae and their usefulness as seed cultures for diverse FPW-derived biogas production. However, biogas yields from FPWs by the subcultured microflorae have not reached their theoretical maximal value. Increasing the surface area-to-volume ratio of the FPWs particles through physicochemical treatments, coupled with a reduction in biogas pressure in the fermentation reactor, could enhance solubilization and accelerate biogas production, which should be investigated in future studies.

## Figures and Tables

**Table 1:** The total organic carbon (TOC, mg/L), total nitrogen (TN, mg/L), and pH value of autoclaved FPW extract.

Food Waste	TOC	TN	TOC/TN Ratio	pH
Cattle bone	87.8	27.5	3.2	8.3
Fish bone	66.3	20.9	3.2	5.8
Carrot peel	230.7	3.8	60.7	5.3
White radish peel	230.0	16.3	14.1	5.0
Cabbage stem	233.5	15.6	14.9	5.4
Lotus stem peel	236.9	10.1	23.5	6.8
Apple core and peel	318.6	1.9	170.8	4.8
Orange peel	311.5	7.1	44.2	4.4
Grape peel	206.1	4.0	51.9	4.5
Rice hull	18.4	0.5	37.3	6.6
Rice bran	147.1	13.1	11.2	6.6
Wheat bran	104.1	6.4	16.2	6.4
Soy sauce lees	62.9	11.1	5.7	5.9
Spent bonito flakes	20.1	6.7	3.0	6.1
Spent dried kelp	156.9	4.2	37.5	6.5
Spent tea leaves	103.4	6.4	16.2	5.5
Spent coffee ground	62.7	5.0	12.6	5.3
Rapeseed oil cake	96.0	5.6	17.0	5.9

## Data Availability

The data presented in this study are available upon request from the corresponding author.

## Supplementary Materials

The following supporting information can be downloaded at: https://www.mdpi.com/article/10.3390/microorganisms11092321/s1, Figure S1: Biogas yield of the subcultures originating from DABYS-A (a), DABYS-B (b), DABYE-G (c), DABYE-S (d), and DABYE-R (e) seed microflorae; Table S1: Biogas production from FPWs by the subcultured DABYS-A microflorae; Table S2: Biogas production from FPWs by the subcultured DABYS-B microflorae; Table S3: Biogas production from FPWs by the subcultured DABYS-G microflorae; Table S4: Biogas production from FPWs by the subcultured DABYS-S microflorae; Table S5: Biogas production from FPWs by the subcultured DABYS-R microflorae.
