# Supplementary material for: The Potential of Digested Sludge-Assimilating Microflora for Biogas Production from Food Processing Wastes
Source: Microorganisms. 2023 Sep 15;11(9):2321. doi: 10.3390/microorganisms11092321 (PMC10535770; doi:10.3390/microorganisms11092321)
Supplement: Supplementary file 1 [file microorganisms-11-02321-s001.zip › microorganisms-2557346-supplementary.pdf]

Table S1. Biogas production from FPWs by the subcultured DABYS-A microflorae

Substrate	Gas	Biogas yield in each subculture (mL/g-substrate)												Biogas composition (%)
		1st subculture	2nd subculture	3rd subculture	4th subculture	5th subculture	6th subculture	7th subculture	8th subculture	9th subculture	10th subculture	Average	Stdev	
Cattle bone	Methane	12.3	10.4	9.1	9.0	9.3	6.3	6.7	7.4	6.8	7.3	8.4	1.9	46.3% CH_4
	Hydrogen	0.0	0.0	0.0	0.0	0.0	0.2	0.0	0.0	0.2	0.2	0.1	0.1	0.4% H_2
	Carbon dioxide	11.5	10.3	10.2	10.3	10.3	9.0	7.1	9.9	9.2	9.5	9.7	1.2	53.3% CO_2
Fish bone	Methane	12.5	9.4	9.8	6.9	8.0	8.0	9.6	10.3	10.1	9.7	9.4	1.5	51.8% CH_4
	Hydrogen	0.0	0.0	0.0	0.1	0.1	0.0	0.0	0.2	1.4	0.0	0.2	0.4	1.0% H_2
	Carbon dioxide	8.4	8.7	8.6	9.0	8.8	9.0	6.6	9.1	8.9	8.8	8.6	0.7	47.2% CO_2
Carrot peel	Methane	0.0	0.0	0.0	0.0	0.0	0.0	0.0	0.0	0.0	0.0	0.0	0.0	0.0% CH_4
	Hydrogen	12.6	11.6	9.8	10.2	3.0	5.6	3.7	8.5	8.0	10.0	8.3	3.2	66.8% H_2
	Carbon dioxide	8.3	5.4	4.2	4.4	2.2	2.7	1.6	3.7	3.9	4.8	4.1	1.9	33.2% CO_2
White radish peel	Methane	0.0	0.0	0.0	0.0	0.0	0.0	0.0	0.0	0.0	0.0	0.0	0.0	0.0% CH_4
	Hydrogen	7.8	11.6	8.6	6.5	7.8	5.2	8.3	5.6	7.2	6.6	7.5	1.8	52.2% H_2
	Carbon dioxide	6.1	5.1	8.1	7.6	6.8	6.7	7.8	7.0	6.2	7.6	6.9	0.9	47.8% CO_2
Cabbage stem	Methane	0.0	0.0	0.0	0.0	0.0	0.0	0.0	0.0	0.0	0.0	0.0	0.0	0.0% CH_4
	Hydrogen	12.9	10.2	11.8	10.8	10.5	10.0	12.0	10.8	11.0	11.0	11.1	0.9	56.2% H_2
	Carbon dioxide	7.0	8.0	9.0	10.1	8.9	9.5	10.0	8.9	7.0	7.9	8.6	1.1	43.8% CO_2
Lotus root peel	Methane	0.0	0.0	0.0	0.0	0.0	0.0	0.0	0.0	0.0	0.0	0.0	0.0	0.0% CH_4
	Hydrogen	4.2	1.2	2.4	2.4	2.7	0.2	4.3	4.0	3.7	4.0	2.9	1.4	37.6% H_2
	Carbon dioxide	5.8	6.4	3.9	4.0	4.1	4.7	3.6	5.5	5.1	5.3	4.8	0.9	62.4% CO_2
Apple core and peel	Methane	0.0	0.0	0.0	0.0	0.0	0.0	0.0	0.0	0.0	0.0	0.0	0.0	0.0% CH_4
	Hydrogen	13.9	7.7	9.9	8.5	9.5	9.1	7.1	9.1	9.0	9.0	9.3	1.8	73.9% H_2
	Carbon dioxide	4.8	2.8	3.8	3.8	3.4	3.0	0.5	3.5	3.7	3.4	3.3	1.1	26.1% CO_2
Orange peel	Methane	0.0	0.0	0.0										
	Hydrogen	0.0	0.0	0.0										
	Carbon dioxide	0.0	0.0	0.0										
Grape peel	Methane	0.0	0.0	0.0	0.0	0.0	0.0	0.0	0.0	0.0	0.0	0.0	0.0	0.0% CH_4
	Hydrogen	10.3	9.1	9.1	10.6	11.4	9.1	9.0	10.2	8.2	9.2	9.6	1.0	73.3% H_2
	Carbon dioxide	5.2	3.1	4.1	3.0	2.9	3.3	3.8	2.8	3.7	3.0	3.5	0.7	26.7% CO_2
Rice bran	Methane	0.0	0.0	0.0	0.0	0.0	0.0	0.0	0.0	0.0	0.0	0.0	0.0	0.0% CH_4
	Hydrogen	10.0	11.8	6.9	5.8	5.4	14.6	8.2	13.6	14.3	12.2	10.3	3.5	55.9% H_2
	Carbon dioxide	10.7	9.4	1.6	7.2	8.4	9.9	6.0	8.4	9.8	9.6	8.1	2.7	44.1% CO_2
Wheat bran	Methane	0.0	0.0	0.0	0.0	0.0	0.0	0.0	0.0	0.0	0.0	0.0	0.0	0.0% CH_4
	Hydrogen	7.9	10.8	12.6	13.5	17.1	15.2	15.3	14.7	16.3	14.2	13.8	2.7	60.9% H_2
	Carbon dioxide	8.8	9.9	7.9	8.9	9.6	9.8	8.8	8.5	8.4	7.9	8.9	0.7	39.1% CO_2
Rice hull	Methane	0.0	0.0	0.0	0.0	0.0	0.0	0.0	0.0	0.0	0.0	0.0	0.0	0.0% CH_4
	Hydrogen	1.3	1.3	1.0	1.3	0.7	0.6	0.9	0.6	0.7	1.0	0.9	0.3	64.3% H_2
	Carbon dioxide	0.6	0.5	0.6	0.3	0.7	0.3	0.6	0.6	0.4	0.6	0.5	0.1	35.7% CO_2
Soy sauce lees	Methane	0.0	0.0	0.0	0.0	0.0	0.0	0.0	0.0	0.0	0.0	0.0	0.0	0.0% CH_4
	Hydrogen	1.1	1.9	1.6	1.8	2.0	2.1	2.0	1.8	2.1	2.2	1.8	0.3	44.8% H_2
	Carbon dioxide	2.7	3.0	2.7	2.4	2.7	2.0	2.2	2.0	1.3	1.8	2.3	0.5	55.2% CO_2
Spent bonito flakes	Methane	12.4	12.0	12.0	12.4	11.9	10.3	11.0	11.1	12.0	11.8	11.7	0.7	58.8% CH_4
	Hydrogen	0.1	0.1	0.2	0.3	0.1	0.3	0.2	0.9	0.2	0.3	0.3	0.2	1.3% H_2
	Carbon dioxide	7.1	6.6	7.0	7.5	7.1	8.0	7.8	8.7	9.1	10.3	7.9	1.1	39.9% CO_2
Spent dried kelp	Methane	0.0	0.0	0.0	0.0	0.0	0.0	0.0	0.0	0.0	0.0	0.0	0.0	0.0% CH_4
	Hydrogen	6.7	10.7	10.0	10.5	9.0	8.5	6.5	10.5	9.8	9.1	9.1	1.5	90.7% H_2
	Carbon dioxide	0.7	0.9	0.8	1.0	1.1	0.7	0.3	1.2	1.1	1.5	0.9	0.3	9.3% CO_2
Spent tea leaf	Methane	0.0	0.0	0.0	0.0	0.0	0.0	0.0	0.0	0.0	0.0	0.0	0.0	0.0% CH_4
	Hydrogen	2.9	3.0	2.5	3.1	3.1	3.0	2.7	3.1	2.8	3.0	2.9	0.2	48.9% H_2
	Carbon dioxide	2.8	2.9	3.1	3.1	3.0	2.7	2.6	3.5	3.1	3.7	3.0	0.3	51.1% CO_2
Spent coffee ground	Methane	0.0	0.0	0.0	0.0	0.0	0.0	0.0	0.0	0.0	0.0	0.0	0.0	0.0% CH_4
	Hydrogen	2.1	2.4	3.1	3.3	3.9	2.9	3.2	3.0	2.8	2.2	2.9	0.5	49.7% H_2
	Carbon dioxide	3.0	2.7	3.1	2.6	2.2	4.0	3.1	3.0	2.6	3.0	2.9	0.5	50.3% CO_2
Rapeseed oil cake	Methane	0.0	0.0	0.0	0.0	0.0	0.0	0.0	0.0	0.0	0.0	0.0	0.0	0.0% CH_4
	Hydrogen	14.1	7.4	7.4	8.0	7.7	12.8	6.8	15.8	17.7	16.1	11.4	4.3	54.1% H_2
	Carbon dioxide	12.1	9.8	10.2	9.3	3.4	7.5	5.4	11.0	13.7	14.1	9.7	3.4	45.9% CO_2

Table S2. Biogas production from FPWs by the subcultured DABYS-B microflorae

Substrate	Gas	Biogas yield in each subculture (mL/g-substrate)												Biogas composition (%)
		1st subculture	2nd subculture	3rd subculture	4th subculture	5th subculture	6th subculture	7th subculture	8th subculture	9th subculture	10th subculture	Average	Stdev	
Cattle bone	Methane	12.1	11.5	13.0	10.0	12.6	10.7	13.5	14.2	8.8	8.7	11.5	1.9	63.7% CH_4
	Hydrogen	0.0	0.1	0.0	0.0	0.0	0.2	0.0	0.3	0.1	0.1	0.1	0.1	0.4% H_2
	Carbon dioxide	5.7	6.6	6.1	7.0	6.8	7.5	7.1	6.5	5.6	5.9	6.5	0.6	35.9% CO_2
Fish bone	Methane	11.1	9.5	8.8	10.5	8.4	8.8	8.5	6.8	8.3	8.6	9.0	1.2	57.3% CH_4
	Hydrogen	0.0	0.0	0.0	0.1	0.0	0.0	0.1	0.2	0.3	0.0	0.1	0.1	0.5% H_2
	Carbon dioxide	6.5	7.0	5.9	6.7	6.5	7.3	6.5	6.8	6.8	6.0	6.6	0.4	42.2% CO_2
Carrot peel	Methane	0.0	0.0	0.0	0.0	0.0	0.0	0.0	0.0	0.0	0.0	0.0	0.0	0.0% CH_4
	Hydrogen	7.5	8.4	9.0	9.5	8.7	10.0	7.7	10.3	9.5	9.2	9.0	0.9	74.0% H_2
	Carbon dioxide	4.3	3.0	3.0	3.5	3.0	2.8	3.2	2.1	3.7	3.0	3.2	0.6	26.0% CO_2
White radish peel	Methane	0.0	0.0	0.0	0.0	0.0	0.0	0.0	0.0	0.0	0.0	0.0	0.0	0.0% CH_4
	Hydrogen	6.5	8.1	7.7	6.9	7.3	7.4	6.1	6.3	6.8	6.3	6.9	0.7	50.0% H_2
	Carbon dioxide	7.5	6.9	7.1	6.5	5.5	8.7	7.1	6.7	7.0	6.0	6.9	0.9	50.0% CO_2
Cabbage stem	Methane	0.0	0.0	0.0	0.0	0.0	0.0	0.0	0.0	0.0	0.0	0.0	0.0	0.0% CH_4
	Hydrogen	7.0	6.5	8.0	6.2	6.0	6.9	6.8	7.1	6.7	7.0	6.8	0.5	60.8% H_2
	Carbon dioxide	5.9	5.8	4.8	4.6	4.0	5.1	3.8	3.0	3.1	4.0	4.4	1.0	39.2% CO_2
Lotus root peel	Methane	0.0	0.0	0.0	0.0	0.0	0.0	0.0	0.0	0.0	0.0	0.0	0.0	0.0% CH_4
	Hydrogen	4.3	4.0	2.7	2.9	3.1	3.6	3.1	2.9	2.8	3.2	3.2	0.5	43.5% H_2
	Carbon dioxide	5.3	5.1	4.8	3.9	2.9	4.3	3.9	3.7	4.2	4.2	4.2	0.7	56.5% CO_2
Apple core and peel	Methane	0.0	0.0	0.0	0.0	0.0	0.0	0.0	0.0	0.0	0.0	0.0	0.0	0.0% CH_4
	Hydrogen	10.0	10.7	10.4	3.6	8.5	6.1	5.9	6.3	8.0	7.8	7.7	2.3	65.0% H_2
	Carbon dioxide	5.1	5.3	4.7	4.3	2.5	3.8	3.3	3.8	4.4	4.3	4.2	0.8	35.0% CO_2
Orange peel	Methane	0.0	0.0	0.0										
	Hydrogen	0.0	0.0	0.0										
	Carbon dioxide	0.0	0.0	0.0										
Grape peel	Methane	0.0	0.0	0.0	0.0	0.0	0.0	0.0	0.0	0.0	0.0	0.0	0.0	0.0% CH_4
	Hydrogen	6.2	9.7	11.3	8.5	6.6	8.0	8.3	8.3	7.1	9.4	8.4	1.5	66.5% H_2
	Carbon dioxide	4.2	4.3	4.0	4.1	3.8	5.2	3.7	4.2	4.9	3.6	4.2	0.5	33.5% CO_2
Rice bran	Methane	0.0	0.0	0.0	0.0	0.0	0.0	0.0	0.0	0.0	0.0	0.0	0.0	0.0% CH_4
	Hydrogen	25.8	16.2	14.6	15.4	15.7	15.2	14.3	16.0	13.1	12.0	15.8	3.7	74.7% H_2
	Carbon dioxide	9.1	9.0	8.3	4.8	4.2	3.8	3.8	4.1	3.4	2.9	5.4	2.5	25.3% CO_2
Wheat bran	Methane	0.0	0.0	0.0	0.0	0.0	0.0	0.0	0.0	0.0	0.0	0.0	0.0	0.0% CH_4
	Hydrogen	12.1	18.6	14.1	11.2	15.5	15.3	12.6	11.0	13.9	13.5	13.8	2.3	79.7% H_2
	Carbon dioxide	2.8	4.7	3.3	2.8	4.5	4.2	3.5	2.5	3.6	3.3	3.5	0.7	20.3% CO_2
Rice hull	Methane	0.0	0.0	0.0	0.0	0.0	0.0	0.0	0.0	0.0	0.0	0.0	0.0	0.0% CH_4
	Hydrogen	0.7	1.4	2.6	2.2	0.8	1.0	0.5	0.5	0.2	0.3	1.0	0.8	73.9% H_2
	Carbon dioxide	0.5	0.6	0.2	0.3	0.3	0.5	0.3	0.4	0.1	0.3	0.4	0.1	26.1% CO_2
Soy sauce lees	Methane	0.0	0.0	0.0	0.0	0.0	0.0	0.0	0.0	0.0	0.0	0.0	0.0	0.0% CH_4
	Hydrogen	2.0	0.8	1.3	1.8	1.9	1.7	2.0	2.0	2.0	2.1	1.8	0.4	45.5% H_2
	Carbon dioxide	1.9	2.0	2.1	2.0	2.0	2.6	2.1	2.0	2.0	2.3	2.1	0.2	54.5% CO_2
Spent bonito flakes	Methane	13.4	14.9	13.9	13.7	14.5	13.2	18.8	17.4	12.2	10.8	14.3	2.3	60.6% CH_4
	Hydrogen	0.0	0.0	0.0	0.0	0.2	0.0	0.0	0.1	0.4	0.2	0.1	0.1	0.4% H_2
	Carbon dioxide	8.6	10.2	9.4	9.4	9.6	8.6	10.7	7.5	9.7	8.1	9.2	1.0	39.0% CO_2
Spent dried kelp	Methane	0.0	0.0	0.0	0.0	0.0	0.0	0.0	0.0	0.0	0.0	0.0	0.0	0.0% CH_4
	Hydrogen	9.4	10.7	10.8	11.3	11.3	11.0	11.1	10.5	12.3	11.0	10.9	0.7	76.7% H_2
	Carbon dioxide	3.5	3.7	3.1	3.0	3.1	3.5	3.3	3.3	3.7	3.0	3.3	0.3	23.3% CO_2
Spent tea leaf	Methane	0.0	0.0	0.0	0.0	0.0	0.0	0.0	0.0	0.0	0.0	0.0	0.0	0.0% CH_4
	Hydrogen	4.0	3.2	3.3	4.2	4.9	3.7	4.0	4.1	4.3	4.7	4.0	0.5	48.8% H_2
	Carbon dioxide	4.8	4.4	3.6	3.7	4.0	4.0	4.2	4.6	4.1	4.9	4.2	0.4	51.2% CO_2
Spent coffee ground	Methane	0.0	0.0	0.0	0.0	0.0	0.0	0.0	0.0	0.0	0.0	0.0	0.0	0.0% CH_4
	Hydrogen	4.1	3.5	3.3	3.6	3.1	2.9	3.7	3.9	4.1	3.7	3.6	0.4	47.5% H_2
	Carbon dioxide	4.6	4.1	3.9	3.2	3.6	4.7	3.9	4.0	3.1	4.6	4.0	0.6	52.5% CO_2
Rapeseed oil cake	Methane	0.0	0.0	0.0	0.0	0.0	0.0	0.0	0.0	0.0	0.0	0.0	0.0	0.0% CH_4
	Hydrogen	9.7	14.6	12.8	11.4	11.9	12.0	13.0	11.2	12.6	12.7	12.2	1.3	53.7% H_2
	Carbon dioxide	10.8	11.9	10.8	11.2	9.8	12.7	12.0	10.4	8.8	6.9	10.5	1.7	46.3% CO_2

Table S3. Biogas production from FPWs by the subcultured DABYS-G microflorae

Substrate	Gas	Biogas yield in each subculture (mL/g-substrate)												Biogas composition
		1st subculture	2nd subculture	3rd subculture	4th subculture	5th subculture	6th subculture	7th subculture	8th subculture	9th subculture	10th subculture	Average	Stdev	(%)
Cattle bone	Methane	7.3	7.8	7.5	8.0	7.6	7.4	7.7	7.3	6.6	5.9	7.3	0.6	46.9% CH_4
	Hydrogen	0.1	0.0	0.0	0.0	0.1	0.0	0.2	0.0	0.0	0.1	0.1	0.1	0.4% H_2
	Carbon dioxide	7.6	8.2	8.4	8.4	7.9	8.5	8.4	8.2	7.5	9.1	8.2	0.5	52.7% CO_2
Fish bone	Methane	4.5	4.5	3.2	3.3	3.9	3.9	3.5	3.3	2.8	3.2	3.6	0.6	29.1% CH_4
	Hydrogen	0.0	0.0	0.0	0.0	0.0	0.2	0.2	0.3	0.1	0.4	0.1	0.1	1.0% H_2
	Carbon dioxide	7.9	8.8	9.2	8.2	9.6	9.1	8.5	8.3	9.1	7.9	8.7	0.6	69.9% CO_2
Carrot peel	Methane	0.0	0.0	0.0	0.0	0.0	0.0	0.0	0.0	0.0	0.0	0.0	0.0	0.0% CH_4
	Hydrogen	10.2	9.7	10.5	9.3	9.6	8.9	6.7	11.0	8.9	15.7	10.1	2.3	68.9% H_2
	Carbon dioxide	4.6	4.2	4.6	4.0	4.6	4.0	2.9	4.8	4.4	7.4	4.5	1.1	31.1% CO_2
White radish peel	Methane	0.0	0.0	0.0	0.0	0.0	0.0	0.0	0.0	0.0	0.0	0.0	0.0	0.0% CH_4
	Hydrogen	8.1	7.6	7.6	7.0	6.1	7.6	8.4	7.7	8.0	6.2	7.4	0.8	49.3% H_2
	Carbon dioxide	7.1	8.6	7.6	7.4	6.6	8.6	7.4	7.6	8.6	7.2	7.6	0.7	50.7% CO_2
Cabbage stem	Methane	0.0	0.0	0.0	0.0	0.0	0.0	0.0	0.0	0.0	0.0	0.0	0.0	0.0% CH_4
	Hydrogen	9.3	9.9	8.9	9.2	9.7	9.0	8.6	10.9	10.4	10.0	9.6	0.7	57.8% H_2
	Carbon dioxide	6.8	7.1	6.1	6.7	7.0	7.5	6.7	7.5	7.4	7.0	7.0	0.4	42.2% CO_2
Lotus root peel	Methane	0.0	0.0	0.0	0.0	0.0	0.0	0.0	0.0	0.0	0.0	0.0	0.0	0% CH_4
	Hydrogen	0.0	0.0	0.2	0.2	0.3	0.0	0.0	0.0	0.0	0.0	0.1	0.1	1.1% H_2
	Carbon dioxide	6.0	6.0	6.7	6.1	6.5	8.2	6.8	7.0	7.3	6.1	6.7	0.7	98.9% CO_2
Apple core and peel	Methane	0.0	0.0	0.0	0.0	0.0	0.0	0.0	0.0	0.0	0.0	0.0	0.0	0.0% CH_4
	Hydrogen	13.0	9.2	9.7	11.7	9.1	10.6	10.3	9.3	10.2	10.6	10.4	1.2	74.3% H_2
	Carbon dioxide	4.9	3.5	3.9	2.7	3.6	2.7	3.3	3.8	3.6	4.0	3.6	0.6	25.7% CO_2
Orange peel	Methane	0.0	0.0	0.0										
	Hydrogen	0.0	0.0	0.0										
	Carbon dioxide	0.0	0.0	0.0										
Grape peel	Methane	0.0	0.0	0.0	0.0	0.0	0.0	0.0	0.0	0.0	0.0	0.0	0.0	0.0% CH_4
	Hydrogen	11.5	10.8	11.4	11.7	9.8	8.9	10.1	10.5	11.6	11.0	10.7	0.9	68.8% H_2
	Carbon dioxide	5.7	5.4	4.8	4.5	4.9	5.0	4.0	4.1	5.5	4.8	4.9	0.6	31.2% CO_2
Rice bran	Methane	0.0	0.0	0.0	0.0	0.0	0.0	0.0	0.0	0.0	0.0	0.0	0.0	0.0% CH_4
	Hydrogen	8.9	15.7	14.1	13.8	14.9	15.1	12.3	18.1	13.6	15.9	14.2	2.4	64.8% H_2
	Carbon dioxide	6.1	6.5	8.3	7.5	7.7	8.4	7.5	8.5	7.6	9.1	7.7	0.9	35.2% CO_2
Wheat bran	Methane	0.0	0.0	0.0	0.0	0.0	0.0	0.0	0.0	0.0	0.0	0.0	0.0	0.0% CH_4
	Hydrogen	10.1	14.0	12.6	15.0	11.5	10.8	10.8	15.9	18.2	20.4	13.9	3.5	66.5% H_2
	Carbon dioxide	5.1	7.3	7.9	6.9	6.5	6.1	5.9	5.5	9.5	9.6	7.0	1.6	33.5% CO_2
Rice hull	Methane	0.0	0.0	0.0	0.0	0.0	0.0	0.0	0.0	0.0	0.0	0.0	0.0	0.0% CH_4
	Hydrogen	0.7	0.8	0.9	1.3	0.2	0.8	0.8	0.7	2.6	1.5	1.0	0.7	60.7% H_2
	Carbon dioxide	1.1	0.3	0.5	0.5	0.6	1.7	0.3	0.4	0.4	0.8	0.7	0.4	39.3% CO_2
Soy sauce lees	Methane	0.0	0.0	0.0	0.0	0.0	0.0	0.0	0.0	0.0	0.0	0.0	0.0	0.0% CH_4
	Hydrogen	2.0	2.2	2.0	3.0	1.7	2.0	2.0	1.9	1.9	2.1	2.1	0.3	48.9% H_2
	Carbon dioxide	2.8	2.6	2.0	2.3	1.6	2.0	2.1	2.2	2.2	1.9	2.2	0.3	51.1% CO_2
Spent bonito flakes	Methane	10.4	8.1	7.5	7.6	8.4	8.3	6.5	7.8	8.4	7.3	8.0	1.0	48.5% CH_4
	Hydrogen	0.1	0.1	0.0	0.0	0.0	0.0	0.0	0.2	0.2	0.1	0.1	0.1	0.4% H_2
	Carbon dioxide	9.8	9.3	8.8	7.9	9.1	7.6	8.3	7.3	8.6	8.1	8.5	0.8	51.2% CO_2
Spent dried kelp	Methane	0.0	0.0	0.0	0.0	0.0	0.0	0.0	0.0	0.0	0.0	0.0	0.0	0.0% CH_4
	Hydrogen	9.4	10.5	8.8	9.8	11.4	11.3	9.2	9.5	11.3	11.0	10.2	1.0	75.6% H_2
	Carbon dioxide	3.3	3.3	3.8	3.0	3.5	3.2	3.2	3.1	3.5	3.0	3.3	0.3	24.4% CO_2
Spent tea leaf	Methane	0.0	0.0	0.0	0.0	0.0	0.0	0.0	0.0	0.0	0.0	0.0	0.0	0.0% CH_4
	Hydrogen	3.6	3.4	4.8	4.5	4.0	4.1	3.7	4.6	4.5	3.8	4.1	0.5	49.5% H_2
	Carbon dioxide	4.1	3.8	4.8	3.1	4.9	4.5	4.6	4.8	3.7	3.8	4.2	0.6	50.5% CO_2
Spent coffee ground	Methane	0.0	0.0	0.0	0.0	0.0	0.0	0.0	0.0	0.0	0.0	0.0	0.0	0.0% CH_4
	Hydrogen	3.9	3.8	4.1	4.0	3.8	3.5	4.4	4.1	3.8	4.0	3.9	0.2	55.8% H_2
	Carbon dioxide	3.5	2.8	3.8	3.1	3.0	3.1	3.1	2.6	2.6	3.6	3.1	0.4	44.2% CO_2
Rapeseed oil cake	Methane	0.0	0.0	0.0	0.0	0.0	0.0	0.0	0.0	0.0	0.0	0.0	0.0	0.0% CH_4
	Hydrogen	9.6	11.3	9.6	10.3	10.0	10.4	11.3	9.9	10.4	11.4	10.4	0.7	58.3% H_2
	Carbon dioxide	8.6	8.2	7.5	8.8	7.8	6.9	6.8	6.8	6.5	6.7	7.5	0.8	41.7% CO_2

Table S4. Biogas production from FPWs by the subcultured DABYS-S microflorae

Substrate	Gas	Biogas yield in each subculture (mL/g-substrate)												Biogas composition
		1st subculture	2nd subculture	3rd subculture	4th subculture	5th subculture	6th subculture	7th subculture	8th subculture	9th subculture	10th subculture	Average	Stdev	(%)
	Methane	6.8	4.1	4.0	5.2	6.8	7.2	6.1	6.7	5.9	6.1	5.9	1.1	41.7% CH_4
Cattle bone	Hydrogen	0.0	0.2	0.1	0.0	0.2	0.0	0.0	0.0	0.1	0.2	0.1	0.1	0.5% H_2
	Carbon dioxide	7.1	7.8	7.9	7.4	9.5	8.9	8.2	8.5	8.4	7.8	8.2	0.7	57.8% CO_2
	Methane	5.0	3.8	3.8	3.5	3.9	3.8	4.1	2.7	3.7	3.0	3.7	0.6	31.5% CH_4
Fish bone	Hydrogen	0.0	0.0	0.0	0.0	0.0	0.1	0.0	0.0	0.1	0.2	0.0	0.1	0.4% H_2
	Carbon dioxide	8.0	7.3	8.0	7.6	8.1	8.6	7.5	8.3	8.4	8.9	8.1	0.5	68.1% CO_2
	Methane	0.0	0.0	0.0	0.0	0.0	0.0	0.0	0.0	0.0	0.0	0.0	0.0	0.0% CH_4
Carrot peel	Hydrogen	8.2	6.1	8.7	5.4	5.2	4.9	4.3	4.8	5.0	8.1	6.1	1.6	64.4% H_2
	Carbon dioxide	5.0	3.3	4.0	2.7	3.2	3.0	2.4	2.7	3.0	4.3	3.3	0.8	35.6% CO_2
	Methane	0.0	0.0	0.0	0.0	0.0	0.0	0.0	0.0	0.0	0.0	0.0	0.0	0.0% CH_4
White radish peel	Hydrogen	6.1	6.7	6.6	7.0	6.9	7.3	7.1	7.9	7.6	7.0	7.0	0.5	59.8% H_2
	Carbon dioxide	8.1	3.5	3.8	6.1	4.6	4.9	4.8	3.2	4.4	3.7	4.7	1.5	40.2% CO_2
	Methane	0.0	0.0	0.0	0.0	0.0	0.0	0.0	0.0	0.0	0.0	0.0	0.0	0.0% CH_4
Cabbage stem	Hydrogen	10.0	9.2	9.3	9.6	10.0	9.7	9.4	9.4	9.8	9.4	9.6	0.3	58.8% H_2
	Carbon dioxide	7.8	7.1	6.3	6.7	7.0	6.5	6.7	6.6	6.0	6.5	6.7	0.5	41.2% CO_2
	Methane	0.0	0.0	0.0	0.0	0.0	0.0	0.0	0.0	0.0	0.0	0.0	0.0	0.0% CH_4
Lotus root peel	Hydrogen	0.4	0.7	0.6	0.3	0.3	1.0	0.9	0.5	0.7	0.8	0.6	0.2	10.7% H_2
	Carbon dioxide	6.8	6.0	6.4	5.2	0.0	5.1	5.0	5.0	5.6	5.9	5.1	1.9	89.3% CO_2
	Methane	0.0	0.0	0.0	0.0	0.0	0.0	0.0	0.0	0.0	0.0	0.0	0.0	0.0% CH_4
Apple core and peel	Hydrogen	11.9	10.2	7.2	9.6	9.0	8.3	8.3	9.1	9.1	9.6	9.2	1.3	73.3% H_2
	Carbon dioxide	4.6	3.3	2.5	3.1	3.5	3.2	3.2	3.0	3.4	3.7	3.3	0.5	26.7% CO_2
	Methane	0.0	0.0	0.0										
Orange peel	Hydrogen	0.0	0.0	0.0										
	Carbon dioxide	0.0	0.0	0.0										
	Methane	0.0	0.0	0.0	0.0	0.0	0.0	0.0	0.0	0.0	0.0	0.0	0.0	0.0% CH_4
Grape peel	Hydrogen	8.3	9.3	9.3	10.2	10.0	9.3	9.0	8.1	11.0	11.7	9.6	1.1	74.3% H_2
	Carbon dioxide	4.2	3.7	2.1	3.6	3.4	3.1	3.4	3.2	3.0	3.6	3.3	0.5	25.7% CO_2
	Methane	0.0	0.0	0.0	0.0	0.0	0.0	0.0	0.0	0.0	0.0	0.0	0.0	0.0% CH_4
Rice bran	Hydrogen	6.5	4.1	6.8	7.4	8.3	6.7	8.2	6.2	8.5	5.3	6.8	1.4	60.9% H_2
	Carbon dioxide	6.5	3.5	2.7	4.1	5.1	5.3	4.3	3.4	4.5	4.3	4.4	1.1	39.1% CO_2
	Methane	0.0	0.0	0.0	0.0	0.0	0.0	0.0	0.0	0.0	0.0	0.0	0.0	0.0% CH_4
Wheat bran	Hydrogen	2.3	2.7	4.2	6.8	7.8	7.3	9.2	11.6	10.6	11.2	7.4	3.4	64.0% H_2
	Carbon dioxide	6.5	6.1	5.1	4.6	3.1	2.2	2.4	2.0	5.0	4.5	4.1	1.6	36.0% CO_2
	Methane	0.0	0.0	0.0	0.0	0.0	0.0	0.0	0.0	0.0	0.0	0.0	0.0	0.0% CH_4
Rice hull	Hydrogen	0.6	0.7	0.9	0.1	0.2	0.0	1.1	1.1	1.0	1.2	0.7	0.4	61.3% H_2
	Carbon dioxide	0.6	0.3	0.3	0.4	0.5	0.5	0.4	0.3	0.5	0.4	0.4	0.1	38.7% CO_2
	Methane	0.0	0.0	0.0	0.0	0.0	0.0	0.0	0.0	0.0	0.0	0.0	0.0	0.0% CH_4
Soy sauce lees	Hydrogen	2.1	3.0	2.5	1.9	2.0	2.0	2.8	2.1	2.6	2.0	2.3	0.4	50.1% H_2
	Carbon dioxide	2.3	2.6	3.0	2.5	1.9	1.7	2.0	2.0	2.0	2.8	2.3	0.4	49.9% CO_2
	Methane	5.3	10.7	8.0	7.4	8.6	8.6	7.4	7.4	6.8	4.9	7.5	1.7	47.6% CH_4
Spent bonito flakes	Hydrogen	0.0	0.0	0.0	0.0	0.0	0.0	0.0	0.0	0.1	0.0	0.0	0.0	0.1% H_2
	Carbon dioxide	7.6	8.7	8.0	8.1	8.4	9.1	8.0	8.3	7.8	8.6	8.3	0.4	52.3% CO_2
	Methane	0.0	0.0	0.0	0.0	0.0	0.0	0.0	0.0	0.0	0.0	0.0	0.0	0.0% CH_4
Spent dried kelp	Hydrogen	8.7	8.2	9.2	8.9	8.8	9.1	8.6	9.5	8.6	6.7	8.6	0.8	71.3% H_2
	Carbon dioxide	3.9	3.4	3.5	3.1	3.6	3.5	3.3	3.5	3.5	3.5	3.5	0.2	28.7% CO_2
	Methane	0.0	0.0	0.0	0.0	0.0	0.0	0.0	0.0	0.0	0.0	0.0	0.0	0.0% CH_4
Spent tea leaf	Hydrogen	5.1	4.8	4.7	5.7	5.0	4.7	4.1	4.9	5.0	5.5	4.9	0.4	54.7% H_2
	Carbon dioxide	3.9	3.8	4.0	4.1	4.7	3.7	4.7	4.1	4.1	3.8	4.1	0.4	45.3% CO_2
	Methane	0.0	0.0	0.0	0.0	0.0	0.0	0.0	0.0	0.0	0.0	0.0	0.0	0.0% CH_4
Spent coffee ground	Hydrogen	4.7	4.4	4.6	4.4	4.2	4.0	3.9	4.2	4.0	3.8	4.2	0.3	61.6% H_2
	Carbon dioxide	2.2	2.1	2.9	3.1	2.5	2.8	2.8	3.1	2.7	2.3	2.6	0.4	38.4% CO_2
	Methane	0.0	0.0	0.0	0.0	0.0	0.0	0.0	0.0	0.0	0.0	0.0	0.0	0.0% CH_4
Rapeseed oil cake	Hydrogen	5.8	5.2	3.4	2.6	2.8	3.0	3.2	2.8	5.1	4.3	3.8	1.2	32.4% H_2
	Carbon dioxide	8.2	6.1	5.4	5.3	6.2	9.6	9.2	11.2	9.4	9.1	8.0	2.1	67.6% CO_2

Table S5. Biogas production from FPWs by the subcultured DABYS-R microflorae

Substrate	Gas	Biogas yield in each subculture (mL/g-substrate)												Biogas composition
		1st subculture	2nd subculture	3rd subculture	4th subculture	5th subculture	6th subculture	7th subculture	8th subculture	9th subculture	10th subculture	Average	Stdev	(%)
	Methane	6.0	4.8	5.6	3.9	2.7	3.6	4.5	5.6	5.0	4.9	4.6	1.0	35.0% CH_4
Cattle bone	Hydrogen	0.0	0.0	0.0	0.0	0.1	0.0	0.0	0.0	0.0	0.0	0.0	0.0	0.1% H_2
	Carbon dioxide	7.9	8.7	9.3	8.5	8.0	8.6	9.2	8.8	8.4	8.8	8.6	0.4	64.9% CO_2
	Methane	2.6	2.4	2.5	2.6	2.0	1.8	1.8	2.2	3.1	2.4	2.3	0.4	38.1% CH_4
Fish bone	Hydrogen	0.0	0.0	0.0	0.2	0.2	0.1	0.0	0.1	0.0	0.1	0.1	0.1	1.1% H_2
	Carbon dioxide	3.0	3.1	3.7	3.1	4.4	4.7	4.6	4.4	2.8	3.4	3.7	0.7	60.8% CO_2
	Methane	0.0	0.0	0.0	0.0	0.0	0.0	0.0	0.0	0.0	0.0	0.0	0.0	0.0% CH_4
Carrot peel	Hydrogen	10.2	8.9	9.3	9.9	10.5	9.5	6.8	7.9	8.3	8.2	9.0	1.2	65.9% H_2
	Carbon dioxide	3.6	4.1	3.9	4.2	3.8	3.8	4.3	6.4	6.2	6.0	4.6	1.1	34.1% CO_2
	Methane	0.0	0.0	0.0	0.0	0.0	0.0	0.0	0.0	0.0	0.0	0.0	0.0	0.0% CH_4
White radish peel	Hydrogen	8.1	6.3	6.7	7.2	6.1	7.1	6.7	6.2	7.7	7.8	7.0	0.7	54.3% H_2
	Carbon dioxide	5.1	4.6	6.3	5.4	6.1	5.5	6.5	6.4	6.9	5.9	5.9	0.7	45.7% CO_2
	Methane	0.0	0.0	0.0	0.0	0.0	0.0	0.0	0.0	0.0	0.0	0.0	0.0	0.0% CH_4
Cabbage stem	Hydrogen	11.7	10.7	11.6	12.7	9.1	9.7	10.0	10.6	10.3	10.7	10.7	1.0	64.0% H_2
	Carbon dioxide	5.8	5.9	6.9	7.0	5.8	6.0	5.2	5.0	6.0	6.8	6.0	0.7	36.0% CO_2
	Methane	0.0	0.0	0.0	0.0	0.0	0.0	0.0	0.0	0.0	0.0	0.0	0.0	0.0% CH_4
Lotus root peel	Hydrogen	0.4	0.1	0.0	0.2	0.2	0.0	0.0	0.0	0.0	0.0	0.1	0.1	1.9% H_2
	Carbon dioxide	5.8	5.6	5.5	5.4	3.1	2.8	3.2	3.3	6.4	5.5	4.7	1.4	98.1% CO_2
	Methane	0.0	0.0	0.0	0.0	0.0	0.0	0.0	0.0	0.0	0.0	0.0	0.0	0.0% CH_4
Apple core and peel	Hydrogen	8.3	8.1	9.9	9.2	8.0	5.9	8.8	9.3	6.6	8.0	8.2	1.2	70.9% H_2
	Carbon dioxide	4.8	2.6	4.0	3.5	2.5	3.3	3.2	3.6	2.9	3.3	3.4	0.7	29.1% CO_2
	Methane	0.0	0.0	0.0										
Orange peel	Hydrogen	0.0	0.0	0.0										
	Carbon dioxide	0.0	0.0	0.0										
	Methane	0.0	0.0	0.0	0.0	0.0	0.0	0.0	0.0	0.0	0.0	0.0	0.0	0.0% CH_4
Grape peel	Hydrogen	7.9	7.1	10.5	11.6	9.0	9.9	8.9	10.8	11.2	11.6	9.8	1.6	74.5% H_2
	Carbon dioxide	4.1	2.8	3.8	3.7	2.9	3.0	3.7	3.1	2.8	3.9	3.4	0.5	25.5% CO_2
	Methane	0.0	0.0	0.0	0.0	0.0	0.0	0.0	0.0	0.0	0.0	0.0	0.0	0.0% CH_4
Rice bran	Hydrogen	4.3	4.0	5.2	4.3	7.5	5.5	3.6	3.6	3.0	4.1	4.5	1.3	40.7% H_2
	Carbon dioxide	2.0	2.0	2.2	10.0	8.8	9.9	8.5	8.7	3.5	10.1	6.6	3.6	59.3% CO_2
	Methane	0.0	0.0	0.0	0.0	0.0	0.0	0.0	0.0	0.0	0.0	0.0	0.0	0.0% CH_4
Wheat bran	Hydrogen	11.0	12.2	17.6	17.8	17.5	19.9	21.7	18.9	17.1	21.2	17.5	3.5	67.0% H_2
	Carbon dioxide	7.0	7.1	8.6	9.1	3.5	2.9	10.3	15.2	11.6	10.8	8.6	3.7	33.0% CO_2
	Methane	0.0	0.0	0.0	0.0	0.0	0.0	0.0	0.0	0.0	0.0	0.0	0.0	0.0% CH_4
Rice hull	Hydrogen	1.1	1.5	1.9	1.8	1.9	1.4	1.6	1.5	0.9	1.4	1.5	0.3	61.7% H_2
	Carbon dioxide	1.0	0.9	1.0	1.1	0.6	0.9	1.5	1.0	0.7	0.8	0.9	0.3	38.3% CO_2
	Methane	0.0	0.0	0.0	0.0	0.0	0.0	0.0	0.0	0.0	0.0	0.0	0.0	0.0% CH_4
Soy sauce lees	Hydrogen	2.6	2.3	3.1	3.1	1.0	0.9	1.4	1.8	1.9	1.4	2.0	0.8	49.8% H_2
	Carbon dioxide	1.9	2.9	1.9	2.1	2.0	1.0	2.7	1.8	2.0	1.5	2.0	0.5	50.2% CO_2
	Methane	5.7	10.0	9.4	9.9	7.4	8.5	7.9	7.8	7.5	7.8	8.2	1.3	48.2% CH_4
Spent bonito flakes	Hydrogen	0.1	0.0	0.4	0.3	0.6	0.0	0.1	0.1	0.3	0.2	0.2	0.2	1.2% H_2
	Carbon dioxide	5.9	9.1	8.7	8.9	7.0	8.1	9.6	9.7	10.1	8.7	8.6	1.3	50.6% CO_2
	Methane	0.0	0.0	0.0	0.0	0.0	0.0	0.0	0.0	0.0	0.0	0.0	0.0	0.0% CH_4
Spent dried kelp	Hydrogen	6.4	7.4	8.6	6.6	8.6	9.9	8.0	8.8	10.0	9.6	8.4	1.3	72.7% H_2
	Carbon dioxide	2.7	3.1	2.9	3.5	3.4	3.2	3.2	3.0	3.1	3.5	3.1	0.3	27.3% CO_2
	Methane	0.0	0.0	0.0	0.0	0.0	0.0	0.0	0.0	0.0	0.0	0.0	0.0	0.0% CH_4
Spent tea leaf	Hydrogen	4.2	5.0	4.9	4.8	5.0	5.2	5.8	4.7	4.9	5.0	4.9	0.4	48.7% H_2
	Carbon dioxide	5.2	5.7	5.7	5.0	4.9	5.0	5.5	4.9	5.0	5.0	5.2	0.3	51.3% CO_2
	Methane	0.0	0.0	0.0	0.0	0.0	0.0	0.0	0.0	0.0	0.0	0.0	0.0	0.0% CH_4
Spent coffee ground	Hydrogen	4.4	4.1	4.7	4.0	3.4	4.2	4.1	3.8	4.2	4.0	4.1	0.4	55.7% H_2
	Carbon dioxide	3.2	3.7	3.1	3.2	3.7	3.7	3.2	1.8	3.7	3.1	3.2	0.6	44.3% CO_2
	Methane	0.0	0.0	0.0	0.0	0.0	0.0	0.0	0.0	0.0	0.0	0.0	0.0	0.0% CH_4
Rapeseed oil cake	Hydrogen	12.8	10.2	8.0	9.5	17.5	17.2	19.9	17.5	19.7	19.1	15.1	4.6	55.9% H_2
	Carbon dioxide	11.8	11.2	12.9	12.2	12.2	12.4	11.3	11.9	11.5	11.8	11.9	0.5	44.1% CO_2

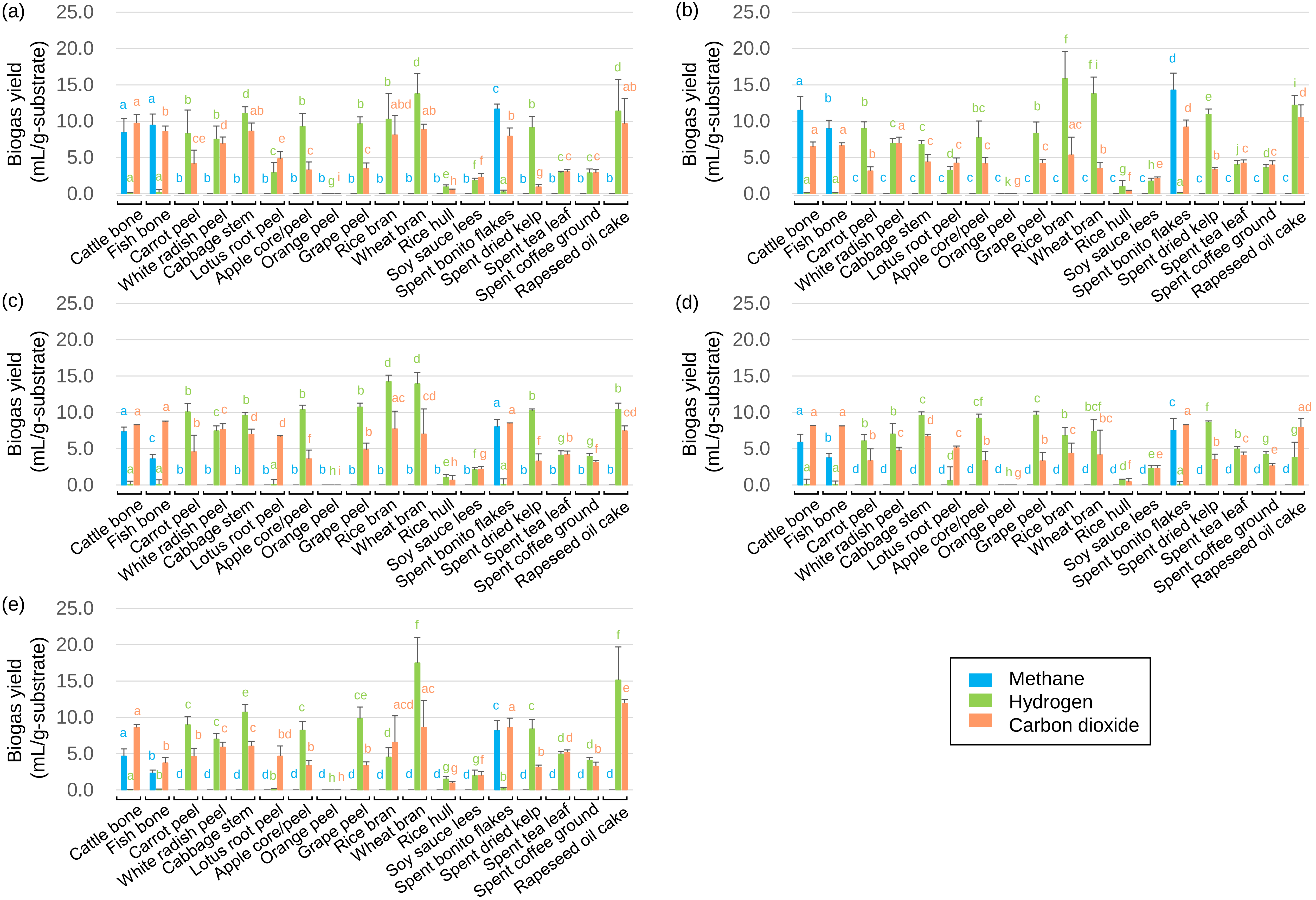

Figure S1. Biogas yield of the subcultures originating from DABYS-A (a), DABYS-B (b), DABYE-G (c), DABYE-S (d), and DABYE-R (e) seed microflorae. The data are presented as the mean ± standard deviation of independent triplicates. The blue, green, and orange colored letters on the columns indicate significant differences at $P < 0.05$ (Student's *t*-test) in methane, hydrogen, and carbon dioxide yield, respectively.
